# *WUSCHEL* Overexpression Promotes Callogenesis and Somatic Embryogenesis in *Medicago truncatula* Gaertn

**DOI:** 10.3390/plants10040715

**Published:** 2021-04-07

**Authors:** Aline Kadri, Ghislaine Grenier De March, François Guerineau, Viviane Cosson, Pascal Ratet

**Affiliations:** 1 UniLaSalle laboratoire de Biotechnologies Végétales, 19 Rue Pierre Waguet, 60000 Beauvais, France; alinekadri@hotmail.com (A.K.); ghislaine.grenierdemarch@grandest.fr (G.G.D.M.); 2 Faculty of Sciences IV, Lebanese University, 1809 Zahle, Lebanon; 3 Région Grand Est, 5 rue de Jéricho, 51037 Châlons-en-Champagne, France; 4 Biologie des Plantes et Innovation (BIOPI), Université de Picardie Jules Verne, 33 rue St Leu, 80039 Amiens, France; francois.guerineau@u-picardie.fr; 5 Laboratoire Génome et Développement des Plantes, UMR5096 CNRS/UPVD, 58 avenue Paul Al-duy, 66860 Perpignan Cedex, France; viviane.jean@univ-perp.fr; 6 CNRS, INRAE, Univ Evry, Institute of Plant Sciences Paris-Saclay (IPS2), Université Paris-Saclay, 91405 Orsay, France; 7 Institute of Plant Sciences Paris-Saclay IPS2, Paris Diderot, Sorbonne Paris-Cité, Bâtiment 630, 91405 Orsay, France

**Keywords:** callogenesis, embryogenic potential, growth regulators, *Medicago truncatula*, somatic embryogenesis, *WUSCHEL*

## Abstract

The induction of plant somatic embryogenesis is often a limiting step for plant multiplication and genetic manipulation in numerous crops. It depends on multiple signaling developmental processes involving phytohormones and the induction of specific genes. The *WUSCHEL* gene (*WUS*) is required for the production of plant embryogenic stem cells. To explore a different approach to induce somatic embryogenesis, we have investigated the effect of the heterologous *Arabidopsis* *WUS* gene overexpression under the control of the jasmonate responsive *vsp1* promoter on the morphogenic responses of *Medicago truncatula* explants. *WUS* expression in leaf explants increased callogenesis and embryogenesis in the absence of growth regulators. Similarly, *WUS* expression enhanced the embryogenic potential of hairy root fragments. The *WUS* gene represents thus a promising tool to develop plant growth regulator-free regeneration systems or to improve regeneration and transformation efficiency in recalcitrant crops.

## 1. Introduction

Somatic embryogenesis is a promising approach and a powerful tool for the mass propagation of plants. This process also provides a potential model to investigate the early regulatory and developmental events in plant embryogenesis [1]. The somatic embryogenesis system is characterized by a sequence of events that includes stimulation of cell proliferation, acquisition of embryogenic competence and induction of embryogenesis. Explant cells can be induced to an embryogenic state by a variety of procedures that usually include exposition to exogenous stimuli like plant growth regulators, certain stress conditions (pH shock, low or high temperature, osmotic shock, drought) or treatments with various chemical substances [2,3]. In response to these signals, somatic cells acquire an embryogenic competence resulting from the action of a complex signaling network and from the reprogramming of gene expression patterns.

Studies of factors and genes controlling in vitro plant morphogenesis are important for the development of improved regeneration systems and for the analysis of molecular mechanisms underlying plant embryogenesis. Genes regulating the plant stem cell development have been identified, like the *Arabidopsis LEAFY COTYLEDON* genes (*LEC1*, *LEC2)*, *FUSCA3* (*FUS3*), *SOMATIC EMBRYOGENESIS RECEPTOR KINASE 1 (SERK1), BABY BOOM (BBM)* and *WUSCHEL* (*WUS*) playing key roles in controlling embryo development [4,5,6,7]. Similarly, several transcription factors were used to induce ectopic formation of somatic embryos in *Arabidopsis.* These include *LEAFY COTYLEDON* genes (*LEC1, LEC2* and *LEC1-LIKE), WUSCHEL* (*WUS*), *PLANT GROWTH ACTIVATOR 37* (*PGA 37*) and *EMBRYOMAKER (EMK)* gene [8,9,10,11,12]. Similar attempts have been reported in other species using *BABY BOOM (BBM)* in tobacco [13], pepper [14] cacao [15] and rice [16], *LEC1* and *LEC2* in tobacco [17], *AGAMOUS-LIKE15* in soybean [18], *BBM* and *WUS2* in maize [19].

The *WUSCHEL*-related homeobox (*WOX*) gene family is a unique transcription factor family in plants and belongs to the homeobox (HB) superfamily. This *WOX* family is characterized by the phylogenetic relatedness of its homeodomains [20]. The analysis of *WOX* gene expression and function shows that *WOX* family members fulfill specialized functions in key developmental processes in plants, including embryonic development, maintenance of meristematic stem cells, development of lateral organs, seed formation and regeneration of isolated tissues and organs [21,22].

The Arabidopsis *WUS* gene is essential for regulating cell division and differentiation during plant development [7]. *WUS* expression is confined to a small group of cells in the lower part of the central zone of the shoot apical meristem, but can drive signals across cell layers and is expressed non autonomously [23]. The *WUS* gene is required to specify stem-cell identity and to maintain a pool of pluripotent stem cells in the shoot apical meristem (SAM). Thus, *wus* mutants fail to organize a functional SAM. During embryogenesis, *WUS* plays a key role by promoting the vegetative-to-embryonic transition and maintaining the identity of the embryonic stem cells [10,24].

Early expression of *WUS* is characteristic of somatic embryogenesis in *Arabidopsis*, *Medicago* and *Zea* [20,25,26]. *WUS* overexpression has been reported to enhance somatic embryogenesis in species such as *N. tabacum* [27], *Coffea canephora* [28], *Capsicum chinense* [29], *Picea glauca* [30], *Gossypium hirsutum* [31] and more recently in *Medicago truncatula* [32]. *WUS* also promotes the formation of embryogenic calli in *G. hirsutum* [33]. However, *WUS* overexpression can result in abnormal somatic embryos formation and can prevent seedling generation [29,30,33].

In this work, we have investigated the effect of the ectopic overexpression of the *A. thaliana WUS* (*AtWus*) gene expressed from the *vsp1* jasmonate inducible promoter on somatic embryogenesis from *Medicago truncatula* (Gaertn.) leaflets and hairy root segments, in the presence or absence of growth regulators. The use of the jasmonate inducible promoter *vsp1* was explored to ectopically express *WUS.* We studied the effect of *WUSHEL* expression on callogenesis and somatic embryogenesis of plants with the aim of using it as a substitute for plant growth regulators, in the model plant for somatic embryogenesis *M. truncatula*. Remarkably, transgenic tissues (over)expressing *WUS* present an initiation of callogenesis and an increase of embryogenesis even in the absence of growth regulators in the culture medium.

## 2. Results

The effect of *WUSCHEL* overexpression during regeneration via somatic embryogenesis was discernable when comparing *WUS*-expressing explants to control explants transformed with the pCambia-*bar* vector. Developmental and morphological characteristics were observed from the appearance of proembryogenic calli till the development of somatic embryos. The transgenic status of the plants selected by phosphinothricin was confirmed by polymerase chain reaction (PCR) amplification of the *BAR* and *WUS* sequences.

### 2.1. The Designed Construct Allows WUS Gene Expression but Not Induction by Jasmonate

The leaves and root segments transformed by *WUS* as well as those of the control were cultured on media treated with or without jasmonate for callogenesis and embryogenesis induction. In order to follow the expression of the transgene in the explants, we tested *WUS* expression via quantitative real-time polymerase chain reaction (qRT-PCR) in 7- and 14-day-old calli formed in the presence or absence of jasmonate. The experiment showed that the *WUS* relative expression level was not significantly different between transgenic calli produced in the presence or absence of jasmonate on leaf explants (2.7 and 2.4, respectively) and on roots (2.4 and 2.1, respectively) (*p* > 0.05). However, *WUS* calli and embryos showed a significantly higher expression level than the calli transformed with pCambia1301-bar (0.07) used as negative controls demonstrating expression of the transgene. We deduced from this experiment that the vsp1-*WUS* gene was expressed in the presence and in the absence of the inducible agent (jasmonate). Accordingly, no significant differences were observed in the callogenesis and embryogenesis of *WUS* explants (leaves and root segments) cultured on media with or without jasmonate (see below). The results presented below are those obtained for *WUS* explants and controls cultured in the presence of jasmonate.

### 2.2. WUSCHEL Enhances Callogenesis in M. truncatula Leaf Explants

In order to test the effect of the *WUS* gene expression on *Medicago* regeneration, leaf fragments were tested for regeneration on media with SH1 medium (supplemented with 4 mg·L^−1^ 2,4-D and 0.5 mg·L^−1^ BAP) or without plant growth regulators (SH0).

On the media without plant growth regulators (SH0), only *WUS* leaves produced calli while the controls did not produce any callus. Strikingly, these *WUS* leaves began callogenesis after 2 weeks of culture and presented a percentage of callus production reaching 59%. Calli formed small clusters near leaf incisions. This result shows that the expression of the *WUS* gene is alone able to induce cell proliferation in absence of plant growth regulators.

**Figure 1:**
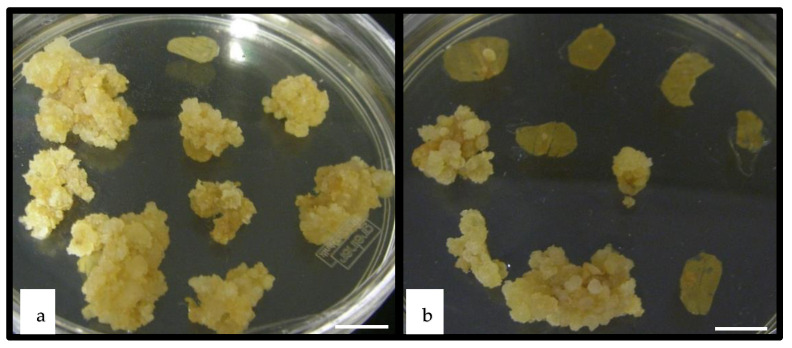
Callus production from *Medicago truncatula* leaf explants. Callus production from *Medicago truncatula* leaf explants transformed with pCambia1301-*WUS-BAR* leaves (**a**) and from leaf explants transformed with pCambia-*BAR* (control, **b**), cultured on SH1 medium (4 mg·L^−1^ 2,4-D, 0.5 mg·L^−1^ BAP) during 3 weeks. Scale bar represent 1.0 cm.

As for the leaf explants cultured on SH1, calli production took place two weeks earlier on *WUS* leaf explants (within 10 days of culture) than on the controls (three weeks of culture). The percentage of explants giving rise to calli was statistically higher in *WUS* transgenic leaves (*p* ˂ 0.1), with 74% of *WUS* leaves producing calli clusters against 46% for the controls (Figure 1). Calli were observed around the edge of the incisions and later covered the entire leaf surface, forming large embryogenic cell clusters. These calli were translucent-whiteand friable [34]. Thus, calli production was also enhanced on *WUS* expressing leaf explants in presence of callus inducing plant growth regulators.

### 2.3. WUSCHEL Enhances Embryogenesis in M. truncatula Leaf Explants

The *WUS* calli produced on SH0 and transferred to embryogenic medium (SH2 without growth regulators) started to produce embryos after three weeks of culture. Only 7.5% of these calli were embryogenic and produced on average 26 embryos per calli.

**Figure 2:**
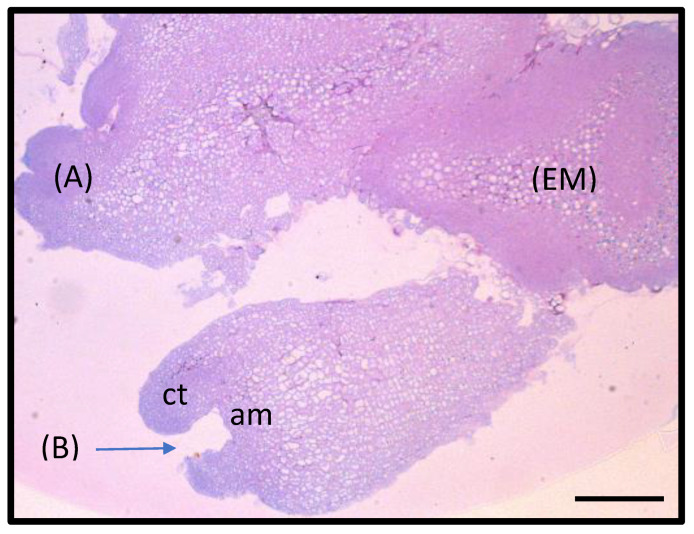
Histological longitudinal section of *WUS* embryogenic calli (EM) after 3 weeks of culture on SH1 medium showing the produced embryos at various stages of development: (**A**) globular stage somatic embryo; (**B**) cotyledonary-stage somatic embryo with two distinct cotyledons (ct) and apical meristem (am). The bar scale represents 200 μm

**Figure 3:**
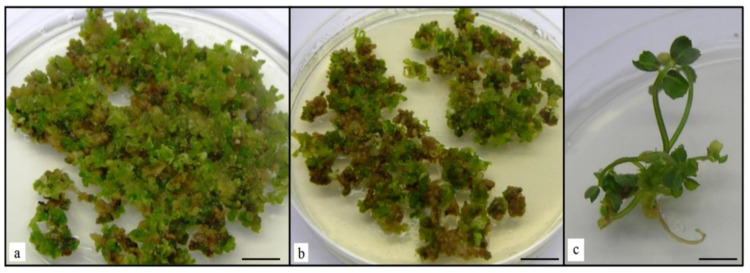
Embryo production from *Medicago truncatula* leaf explants. Embryos production on embryogenic calli formed from *Medicago truncatula* leaf explants transformed with pCambia1301-*WUS-BAR* (**a**) and from leaf explants transformed with pCambia-*BAR* (control) (**b**) after four weeks of culture on SH2 medium (without plant growth regulators). Plantlet developed from a *WUS* embryo of *Medicago* on SH2 medium (**c**). Bar 1.0 cm.

**Figure 4:**
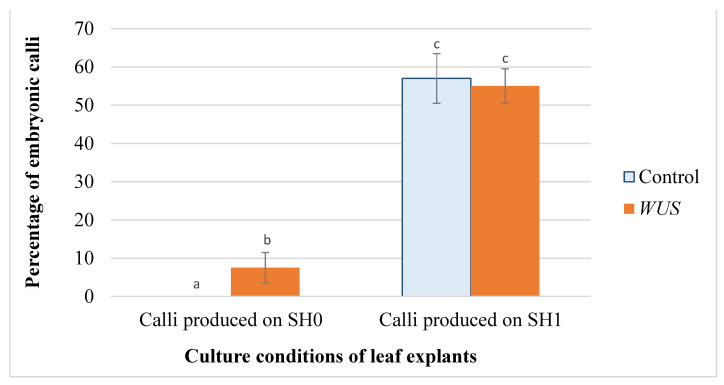
Embryogenic calli production from leaf explants of *Medicago truncatula*. Percentage of embryonic calli formed on leaf explants of *M. truncatula* transformed using pCambia-*BAR* (Control) and pCambia1301-*WUS-BAR* l (*WUS*) cultured on SH2 (without growth regulators). These calli were produced on SH0 (without growth regulators) or on SH1 (4 mg·L^−1^ 2,4-D, 0.5 mg·L^−1^ BAP). For each condition, 60 root explants were cultured per replicate. Three replicates were performed for each condition. Bars with different letters were significantly different at *p* < 0.05.

**Figure 5:**
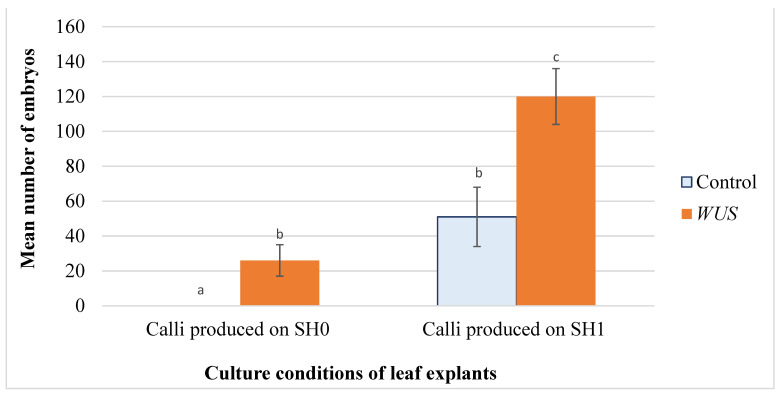
Embryo production from leaf explants of *Medicago truncatula*. Mean number of embryos produced on calli formed leaf explants of *M. truncatula* transformed using pCambia-*BAR* (Control) and pCambia1301-*WUS-BAR* l (*WUS*) cultured on SH2 (without growth regulators). These calli were produced on SH0 (without growth regulators) or on SH1 (4 mg·L^−1^ 2,4-D, 0.5 mg·L^−1^ BAP). Data represent the mean ± standard errors of three replicates. Bars with different letters were significantly different at *p* < 0.05.

As early as two weeks after the transfer of calli cultured on SH1 to embryogenic medium (SH2 without growth regulators), early-stage embryos were produced on cultures transformed with the *WUS* construct. Somatic embryos started to form one week later on the control explants. The histological longitudinal section of a *WUS* embryonic calli showed these embryos of different stage of development (Figure 2). After 4–5 weeks of culture, the percentage of embryogenic calli was not significantly different between *WUS* calli and the controls. In fact, 55% of the *WUS* transgenic calli were embryogenic against 57% for the control explants (Figure 3 and Figure 4). There were also no differences in the production and the phenotype of the embryonic mass between empty vector transformation and non-transformed tissue. However, the average number of embryos produced from each responsive *WUS* calli (120 embryos) was almost 2.3-fold higher than that of control (51 embryos) (Figure 5). Together, these results showed that *WUS* expression allowed embryogenesis from leaf explants cultured in absence of plant growth regulators in the culture medium and increased embryogenesis for leaf explants cultured in the presence of growth regulators.

### 2.4. WUSCHEL Enhances Callogenesis in M. truncatula Hairy Root (HR) Explants

**Figure 6:**
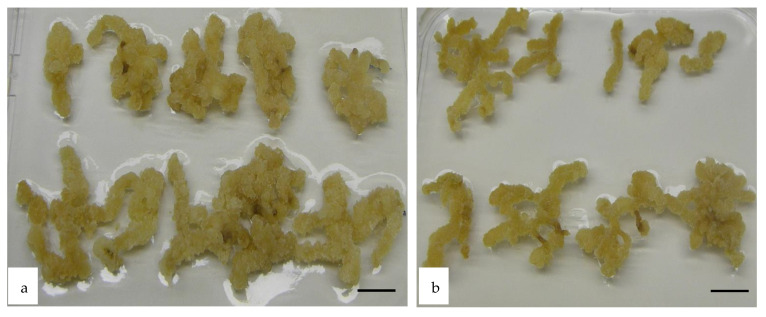
Callus production from *Medicago truncatula* hairy root (HR) explants. Callus production from *Medicago truncatula* leaf explants, transformed with pCambia1301-*WUS-BAR,* two weeks after their transfer to M0 medium (without growth regulator). These calli were produced on a callogenesis medium C (5 mg·L^−1^ 2,4 -D and 0.5 mg·L^−1^ BAP) in the dark for two weeks (**a**) or under photoperiod on M1 medium (50 mg·L^−1^ NAA and 1.5 mg·L^−1^ BAP). Scale bar 1.0 cm (**b**).

In order to test the effect of the *WUS* gene expression on regeneration of *Medicago* hairy root (HR) fragments regeneration, root segments of *M. truncatula* were first cultured under two different regeneration conditions, either directly on M1 medium under photoperiod or first on a callogenesis C medium in the dark for two weeks. The explants from the C medium were then transferred to M0 (without growth regulators) or M1 media. *WUS* and control root explants showed callus production from the first week of culture. Calli were produced along the entire length of the root segments on the two media but the callus sizes of the root segments grown in the dark (C medium) were bigger than those under the photoperiod (Figure 6).

### 2.5. WUSCHEL Enhances Embryogenesis in M. truncatula HR Explants

**Figure 7:**
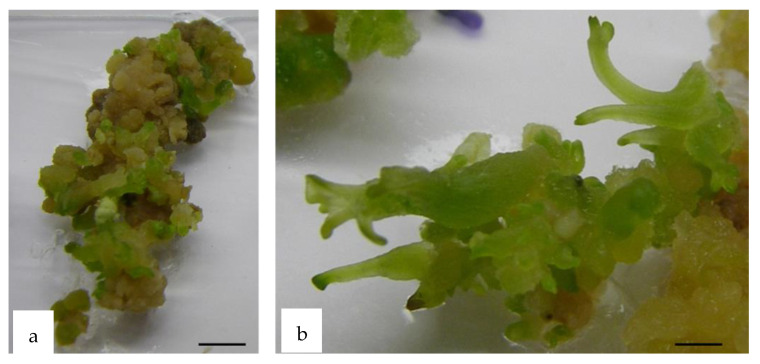
Embryo production from *Medicago truncatula* HR explants. (**a**) *WUS* embryogenic calli produced on root segments of *M. truncatula* after 3 weeks of culture and (**b**) *WUS* somatic embryos at different developmental stages (c: cotyledonary stage and g: globular stage) produced on embryogenic calli after 4 weeks of culture on M0 medium (without plant growth regulators). Bar 1.0 cm (**a**) Bar 0.5 cm (**b**).

**Figure 8:**
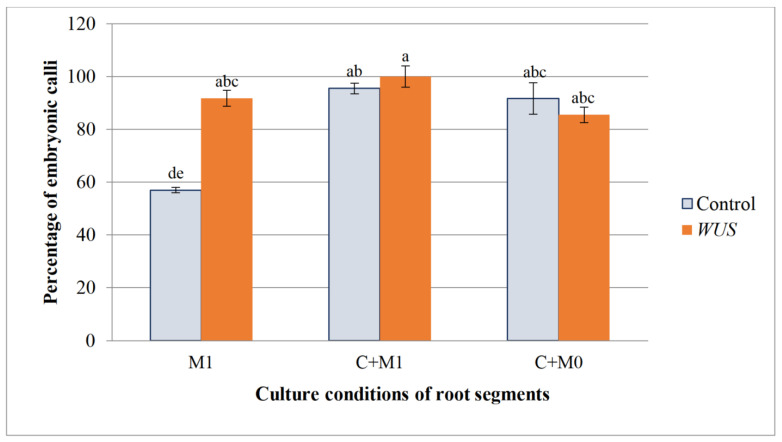
Embryogenic calli production from HR fragments of *Medicago truncatula*. Percentage of embryonic calli formed on hairy root segments of *M. truncatula* transformed by pCambia-*BAR* (Control) and pCambia1301-*WUS*-*BAR* l (*WUS*) cultured in 3 different conditions: directly on M1 medium containing 50 mg·L^−1^ NAA and 1.5 mg·L^−1^ BAP (M1); first, a callogenesis stage on C medium (5 mg·L^−1^ 2,4 -D and 0.5 mg·L^−1^ BAP) and then transferred to M1 medium (C+M1); first a callogenesis stage on C medium and then transferred to M0 medium without growth regulator (C+M0). For each condition, 60 root explants were cultured per replicate. Three replicates were performed for each condition. Bars with different letters were significantly different at *p* < 0.05.

**Figure 9:**
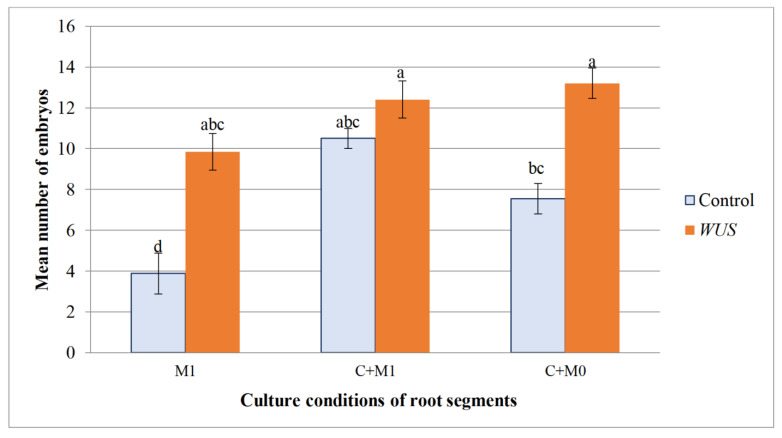
Embryo production from HR fragments. Mean number of embryos produced on calli formed on hairy root segments of *M. truncatula* transformed by pCambia-*BAR* (Control) and pCambia1301-*WUS-BAR* l (*WUS*) cultured in 3 different conditions: directly on M1 medium containing 50 mg·L^−1^ NAA and 1.5 mg·L^−1^ BAP (M1); first, a callogenesis stage on C medium (5 mg·L^−1^ 2,4 -D and 0.5 mg·L^−1^ BAP) and then transferred to M1 medium (C+M1); first a callogenesis stage on C medium and then transferred to M0 medium without growth regulator (C+M0). Data represent the mean ± standard errors of three replicates. Bars with different letters were significantly different at *p* < 0.05.

Calli produced on HR segments grown directly on M1 medium under photoperiodic conditions started to produce proembryos during the second week of culture. After 5 weeks of culture, *WUS* calli showed a significantly higher embryogenic percentage (92%) than controls (57%). In addition, *WUS* calli produced a higher mean number of embryos (9.8 embryos per explant) as compared to the controls (3.8 embryos/explant; Figure 7, Figure 8 and Figure 9). Thus, the expression of the *WUS* gene strongly enhances embryogenesis in these HR fragments in the absence of the callogenesis step. 

For the explants first cultured two weeks in the dark on the callogenesis C medium, embryogenesis did not start until a week after the calli were transferred to M0 or M1 media under the photoperiod. Both controls and *WUS* calli developed a high percentage of embryogenesis. Under these two culture conditions (M0, M1), the percentage of embryonic calli was not significantly different between *WUS* calli and the controls (respectively 100% and 95.5% on M1; 85.5% and 91.7% on M0) (Figure 8). Similarly, *WUS* and control calli produced a high number of embryos (12.4 and 10.5 embryos respectively) following their transfer on M1. In contrast, on the hormone-free M0 medium the number of embryos produced per *WUS* callus (13.2 embryos) was higher than that of the control (7.5 embryos) (Figure 9). It should be noted that under these three culture conditions, *WUS*-expressing HR explants maintained a high number of embryos per explants, whereas the controls showed a significant decrease of the embryos’ mean number when they were cultured directly on M1 or M0 media after a callogenesis step. Again, the expression of the *WUS* gene favored embryogenesis in the presence or absence of plant growth regulators.

### 2.6. Embryo Development and Plantlet Production

The *WUS* and control embryogenic calli followed the typical stages of embryogenesis observed in *Medicago truncatula* [34,35]. They presented a proliferation of embryonic clumps and differentiation of somatic embryo simultaneously (Figure 7). Less than 25% of the produced somatic embryos, from both *WUS* and *BAR* transgenic embryos, were abnormal and later degenerated. Both *WUS* and control somatic embryos produced normal plantlets after being transferred to maturation medium (1/2 MS medium). These plantlets did not show any morphological abnormalities even though they expressed the transgenic *WUS* gene in their leaves (not shown).

## 3. Discussion

Stem cell induction and somatic embryo development involve complex mechanisms including the central role of growth regulators and transcriptional regulators. Mendez-Hernandez et al. [2] emphasized the interactions between the different plant growth regulators during the induction of somatic embryogenesis. Rose [36] suggested the presence of connections between specific plant growth regulators and up-regulated genes during early phases of somatic embryogenesis in *M. truncatula*. The *WUS* gene is essential for somatic embryogenesis and it is designated as being a primary candidate for a gene promoting regeneration in a wide range of species [10,37]. Overexpression of *WUS* has indeed been found to improve the embryonic potential in transgenic plants [10,24,32]. In this work, we used a different approach to induce somatic embryogenesis in order to overcome the hormone dependence of this process. For this, we studied the effect of *WUS* ectopic expression on the somatic embryogenesis capacity of *M. truncatula* (Gaertn.) leaf and hairy root explants under different tissue culture conditions, including plant growth regulators free tissue culture conditions.

Previous studies have shown that the continuous overexpression of *WUS* resulted in malformations and growth alteration [10,33]. Moreover, the level of *WUS* transgene expression was related to the frequency of somatic embryos with aberrant phenotype in white spruce [30]. Similarly, calli produced on *WUS* expressing tobacco explants maintained in induced condition, darkened and the frequency of regeneration was reduced [37]. An inducible system based on the estrogen 17- β -estradiol was used to trigger the expression of *WUS* in different plants [10]. However, somatic embryos formed in the absence of the estrogen in coffee and white spruce [28,30]. In our study, we planned to use a jasmonate-inducible *WUS* gene expressed from the *vsp1* promoter. The aim was to transitory express *WUS* in order to trigger a developmental switch to embryonic callogenesis. Jasmonate was supposed to induce ectopic expression of *WUS* without affecting the in vitro development and growth of transgenic *M. truncatula* explants and embryos. However, in our experiment, *WUS* gene expression was also observed in the absence of jasmonate. According to Arroyo-Herrera et al. [28] using a 17-β-estradiol inducible system, the transcription could be attributed to the position effect of the T-DNA insertion or to a cross reactivity of the inducer receptor (estrogen) interacting with an estrogen-like endogenous molecule. Similar position effect or production of jasmonate in tissue culture can explain our results. The constitutive expression of *WUS* could be due to the influence of the 35S promoter enhancer located next to the *vsp1* promoter in the plasmid construct. *WUS* expression could be also induced by endogenous jasmonate produced as a response to the stress subjected to explants cultured in vitro.

Plant growth regulators are required for the induction of somatic embryogenesis from cultured explants in the majority of plants [38]. In many in vitro culture systems and especially for *Medicago* leaf culture, auxin is required as a pulse to induce callus production. High levels of auxin in the culture medium promote cell proliferation and embryogenic callus formation. The produced calli are then transferred to auxin-free medium for the formation of somatic embryos [34,39]. In our study, the expression of *WUS* induced spontaneous embryogenic calli from *Medicago* leaves grown on plant growth regulators -free basal medium. Only *WUS-*expressing leaves produced embryogenic calli in the absence of growth regulators while leaves transformed with the empty vector did not produce any callus cluster. This is consistent with Chen et al. [25] who suggested that *WUS* is associated with the production of totipotent cells, similar to the way it is involved in stem cell formation and maintenance. Similarly, leaves-derived calli expressing *WUS* produced a higher percentage of embryogenic calli and showed a 2.3-fold improvement (120 embryos versus 51) in somatic embryo production as compared to control, on media without growth regulators. These experiments showed that *WUS* transgenic embryos could be obtained from *Medicago* leaves explants without the use of growth regulators. Zuo et al. [10] suggested that *WUS* can reprogram cell identity, bypassing the auxin requirement or simply taking advantage of the endogenous auxin flux. However, Gallois et al. [24] reported that ectopic expression of *WUS* in roots generated embryo-like structures only in the presence of auxin. In *Arabidopsis thaliana, WUS* transient overexpression caused highly embryogenic callus formation in the presence of auxin [10]. The expression of *AtWUS* induced calli formation as well as a 400% increase in somatic embryo production in coffee plants and a threefold increase in cotton explants in the presence of plant growth regulators [28,31]. Recently, Tvorogova et al. [32] also reported that the overexpression of *MtWOX9-1*, a *WUSCHEL*-related homeobox transcription factor, led to an increase in embryogenic capacity of calli produced from leaf explants in *Medicago.* However, these calli were produced on a callogenesis-inducing medium in the presence of plant growth regulators before being transferred to a plant growth regulator-free medium.

Hairy root (HR) cultures show interesting features such as high growth rate on hormone-free media and genetic stability. HR cultures have also shown promising biosynthetic ability as producers of various biologically active substances and provided insights into root metabolism [40,41]. Moreover, *A. rhizogenes*-mediated hairy root transformation systems are suitable for species recalcitrant to transformation by *A. tumefaciens*, as higher transformation efficiencies are obtained in comparison to *A. tumefaciens*-mediated transformation systems [42]. They may also help faster production of mutants using the CRISPR/CAS9 technology [43]. With the aim of enhancing HR callogenesis and embryogenesis, we studied the effect of *WUS* expression on HR. HR regeneration however requires specific protocols [35]. Using the HR explants with standard in vitro culture protocol, *WUS* and control *Medicago* explants showed similar embryogenesis potential. However, under culture conditions that normally do not support embryogenesis (without the passage through a callogenesis phase or in absence of growth regulators in the embryonic media), *WUS* calli produced a high number of embryos per explant, while the number of embryos produced by control calli was very low. This result showed that somatic embryos can be produced on explants expressing *WUS*, bypassing the need for callus initiation and maintenance.

Chen et al. [25] found that during callogenesis, the clusters of cells expressing *WUS* were the source of cells that formed embryos. Moreover, the ectopic expression of *WUS* resulted in up regulation of other embryogenic regulators such as *LEC1*, *LEC2*, and *FUS3* [44]. Identifying these regulators and elucidating their exact roles in embryogenesis will advance the molecular understanding of plant embryogenesis.

## 4. Materials and Methods

### 4.1. Construction of Binary Plasmids

**Figure 10:**
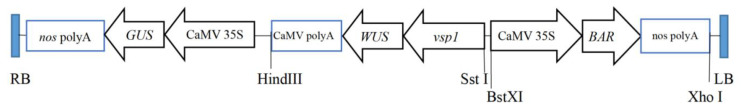
Organization of the T-DNA of plasmid pCambia1301-*WUS*-*bar*. Arrows represent promoters and coding sequences. Rectangles represent polyA regions. Restriction sites used for the construction are also indicated.

Two binary vectors were generated, pCambia1301-*bar* and pCambia1301-*WUS-bar.* These plasmids contain the reporter gene (*GUS*) and the plant selectable marker gene conferring resistance to phosphynothricin (*BAR)*, both expressed from the CaMV35S promoter. In addition, pCambia1301-*WUS-bar* contains the *WUSCHEL* gene under control of the *vsp1* jasmonate inducible promoter (Figure 10).

The vector pCambia1301-*bar* was generated by using pCambia 1301/Pubi-*bar*-Tnos (provided by AFOCEL, Nangis, France) containing the *BAR* gene under the control of the *Pubi* promoter [45]. To place the *BAR* gene under control of the CaMV35S promoter, the *BAR*::Tnos was recovered from pCambia 1301/Pubi-bar-Tnos and inserted into the pBLTI221 vector [46] under the control of CaMV35S promoter using *Bam*HI and *Eco*RI. Restriction sites *Bst*XI and *Xho*I were present on either sides of the cassette CaMV35S-*bar-Tnos.* The *hpt* gene was removed from pCAMBIA1301 (Center for the Application of Molecular Biology to the International Agriculture of Canberra, Australia) by digesting the plasmid with *Bst*XI/*Xho*I and the CaMV35S: *bar* gene, was ligated into the digested pCambia1301 plasmid, between the *Bst*XI and *Xho*I sites.

As a first step to construct pCambia1301-*WUS-bar*, the *WUS* coding sequence was amplified from cDNAs synthesized from RNA extracted from *Arabidopsis thaliana* Columbia stem tips carrying flower buds, flowers and young siliques, using the 5’-GAGCTGCAGAACAATGGAGCCGCCACAGC-3’ and 5’-GAGGAATTCTAGTTCAGACGTAGCTC-3’ primers. The PCR product was inserted into pGEM^®^-T Easy (Promega) and the *WUS* sequence was verified by nucleotide sequencing. To place the *WUS* coding sequence under the control of the *vsp1* promoter, an expression cassette was constructed as follows. The *vsp1* promoter, recovered from pAM35 [47] as an *Sst* I-*Pst* I fragment, was inserted into pJIT117 [48] in place of the 35S promoter, generating pAM44. The *WUS* coding sequence was inserted into pAM44 using *Pst* I and *Eco*RI, generating p44wus. In this construct, the *WUS* coding sequence is placed between the *vsp1* promoter and the CaMV polyadenylation signal. The *Bgl* II fragment of p44wus carrying the *vsp1*-*WUS*-CaMV polyA fusion was inserted into the *Bam*HI site of pUC19, generating pUC44wus. The gene fusion was then moved into the binary vector pCambia 1301 bar using *Sst* I and *Hin*dIII, generating pCambia1301-*WUS-bar.* Molecular cloning was performed according to standard methods [49].

### 4.2. Bacterial Strains and Growth Conditions

The pCambia1301-*bar* and pCambia1301-*WUS-bar* vectors were separately transferred into *Agrobacterium tumefaciens* strains AGL0 for *M. truncatula* leaf transformation and into *Agrobacterium rhizogenes* strain A4Tc24 for *M. truncatula* hairy root transformation, using a CaCl_2_ method [50]. For each condition, cultures of *A. tumefaciens* were initiated from a single colony or from glycerol stocks and grown overnight at 28 °C with shaking (150 rpm) in liquid YEB medium [51] containing 50 mg·L^−1^ kanamycin, to mid log phase (OD_600_ = 0.9–1.2). For leaf coculture, the *A. tumefaciens* cells strain AGL0 were collected by centrifugation and resuspended in SH based liquid inoculation medium [52]. For radicle coculture, a single resistant colony of A4Tc24 was streaked on solid YEB medium containing 100 mg·L^-1^ kanamycin and incubated at 28 °C for two days.

### 4.3. Agrobacterium tumefaciens-Mediated Leaf Transformation of M. truncatula

*Agrobacterium tumefaciens*-mediated transformation and regeneration via somatic embryogenesis of *M. truncatula* was done as described by Trinh et al. (1998) and Cosson et al. (2015) [34,39]. Plantlets of *M. truncatula* (Gaertn.) line R108-1 (c3) [39,53] were grown in Magenta boxes containing half strength of SH based media [52]. The plantlets were cultured under a 16 h light (200 μE/m^2^/s)/8h dark photoperiod at 24 ± 2 °C. Leaves from 2- to 3-week-old in vitro plantlets were cut off and each foliole was wounded with 3 to 4 scalpel cuts.

Leaf explants were transferred into two flasks, one containing a suspension of AGL0 cells carrying pCambia1301-*bar* and the other containing AGL0 with pCambia1301-*WUS-bar*. A vacuum (760 mm Hg) was generated in the flasks for 20 min using a tap water pump.

### 4.4. Coculture and Culture Media of M. truncatula Leaves

The infected explants were placed adaxial side facing up on the SH0 and SH1 coculture media. These media are based on SH media (N6 macroelements, SH microelements, SH vitamins, 100 mg·L^−1^ *myo*-inositol, 30 g·L^−1^ sucrose, and 3 g·L^−1^ phytagel (Sigma-Aldrich, Saint-Louis, Missouri, USA), pH 5.8) and differ respectively by the absence of plant growth regulators (SH0) or the presence of 2,4-D at 4 mg·L^−1^ and of BAP at 0.5 mg·L^−1^ (callogenic medium SH1). For each condition, 60 explants were incubated in the dark at 20 °C for 2 days and then cultured in the dark on selection SH0 and SH1 media supplemented with augmentin at 800 mg·L^−1^ to suppress *Agrobacterium* growth and with phosphinothricin at 3 mg·L^−1^ to select transformed plant cells. 

After 5 weeks of culture, explants and their developing calli were transferred onto SH2 embryonic medium (SH0 medium with 20 mg·L^−1^ of sucrose and without plant growth regulators), supplemented with augmentin at 800 mg·L^−1^ and phosphinothricin at 3 mg·L^−1^. The explants (10 explants per Petri dish) were cultivated at 24°C under a photoperiod of 12 h (75–110 μE m^−2^ s^−1^).

### 4.5. Agrobacterium rhizogenes-Mediated Hairy Root Production in M. truncatula

*Agrobacterium rhizogenes*-mediated transformation was done as described by Boisson-Dernier et al. (2001) [54]. Scarified and surface sterilized seeds of *M. truncatula* (Gaertn.) line R108-1 (c3) [53] were germinated on inverted agar plates at 14°C in the dark. After 2 days of germination, the radicle was sectioned approximately 3 mm from the root tip with a sterile scalpel. Sectioned radicles were inoculated by coating the freshly cut surface with *A. rhizogenes* A4Tc24 carrying pCambia1301-*bar* or pCambia1301-*WUS-bar* grown on YEB solid medium [51]. The inoculated sectioned seedlings were then placed on Fahraeus medium [55] in square Petri dishes (12 × 12 cm). The Petri dishes containing the inoculated seedlings (10 seedlings per Petri dish) were placed at an angle of approximately 45° in a growth chamber at 20 °C for one week (16 h light/8 h dark photoperiod and a light intensity of 75 μE m^−2^ s^−1^). After 7 days of co-culture, seedlings were transferred onto ½ MS medium [56] supplemented with Augmentin at 400 mg·L^−1^ for HR development.

### 4.6. Culture Media of M. truncatula Hairy Roots

For HR regeneration, hairy root fragments of approximately 1 to 2 cm in length were excised and sub-cultured every 2 weeks in horizontal Petri dishes under different culture conditions:

(a) Directly on MS medium [56] containing 50 mg·L^−1^ NAA and 1.5 mg·L^−1^ BAP (M1 medium) under a 16 h light/8 h dark photoperiod.

(b) On a callogenesis inducing MS medium [56] containing 5 mg·L^−1^ 2,4-D and 0.5 mg·L^−1^ BAP (C medium) for two weeks in the dark. Then, the produced calli were transferred to two media: MS medium without plant growth regulators (medium M0) or MS medium containing 50 mg·L^−1^ NAA and 1.5 mg·L^−1^ BAP (medium M1) and placed under a 16 h light/8 h dark photoperiod.

### 4.7. Induction of WUS Transgene Expression

For enhancing *WUS* expression, sterile capsules containing 10 mM of methyl jasmonate (100 μL) were placed in each Petri dish. Explants were treated with jasmonate from the first day of culture till the production of the first proembryos, i.e., for at least two to three weeks.

### 4.8. Molecular Analysis, Polymerase Chain Reaction (PCR) Analysis

Total genomic DNA was isolated from leaves and roots of transformed plants and control plants using the DNeasy plant Mini Kit (Qiagen, Les Ulis, France) according to the manufacturer recommendations. Polymerase chain reaction (PCR) amplification was performed in a 20 μL reaction volume consisting of 10X buffer (Promega, Madison, WI, USA), 50 mM KCl, 1.5 mM MgCl_2_, 100 mM dNTPs, 0.5 U Taq DNA polymerase (Promega, Madison, WI, USA), 250 nM primers, and 20 ng template DNA. The primers 5′-GCCATTTGAAGCCGATGTCACGCC-3′ and 5′-GTATCGGTGTGAGCGTCGCAGAAC-3′ were used to amplify a 1050 bp *GUS* fragment. The primers 5′-CTACATCGAGACAAGCACGGTCAA-3′ and 5′-GCTGAAGTCCAGCTGCCAGAAA-3′ were used to amplify a 427 bp *BAR* fragment. The primers 5′-CCGCCACAGCATCAGCATCAT-3′ and 5′-CACCACATTCAGTACCTGAGCT-3′ were used to amplify a 529 bp the *WUS* fragment. Cycling parameters for *BAR* and *WUS* amplification began with an initial denaturation at 94 °C for 2 min, followed by 30 cycles of denaturation (94 °C for 2 min), annealing (55 °C for 30 s) and extension (60 °C for 30 s), then a final extension at 72 °C for 5 min. The cycling parameters for *GUS* amplification were similar except that the annealing temperature was 60 °C. PCR amplification products were analyzed by electrophoresis in 1% agarose gels. 

### 4.9. Real-Time PCR (qRT-PCR) Analysis

*WUS* expression was detected by RT-PCR. Total RNA was extracted from calli and embryos of *M. truncatula* plants transgenic for *WUS* at different time points using the RNeasy Mini Kit (Qiagen, Les Ulis, France) according to the manufacturer instructions. cDNA synthesis was performed using the QuantiTect^®^ Reverse Transcription (Qiagen, Les Ulis, France) with integrated removal of genomic DNA contamination. Real-time PCR was performed using the QuantiTect^®^ SYBR^®^ Green PCR (Qiagen, Les Ulis, France). Primers 5′-ATCCAGAAGATCACTGCAAG-3′ and 5′-TGGTGTAAATTGGTTCTCTTAGTAAT-3′ were designed to quantify the *WUS* expression. Normalization was done using the *GLYCERALDEHYDE-3-P DEHYDROGENASE* (*GADPH*) gene using the primers 5′-ACAAACATGGGAGCATCCTTACTAG-3′ and 5′-GTTTTTACCGACAAGGACAAAGCT-3′. Reverse transcription was performed at 50 °C for 30 min, followed by PCR activation at 90 °C for 15 min and then 25 cycles of PCR amplification (94 °C for 1 min, 52 °C for 1 min and 72 °C for 1 min). Transcript abundance was estimated using the comparative threshold cycle (Ct) method and was calculated as *2*^−ΔΔCt^, where ΔΔCt = (Ct_target_ − Ct_GAPDH_)_Time x_ − (Ct_target_ − Ct_GAPDH_)_Calibrator._

### 4.10. Histology and Microscopy

For embryo observation, samples of embryogenic calli were fixed in a mixture of 3.5% (*w*/*v*) of glutaraldehyde, 0.2 M of cacodylic acid and 2.6% (*w*/*v*) of sucrose, and then dehydrated in an ethanol series (15%, 30%, 50% for 5 min each and 70%, 90%, 100% for 2 h each). The fixed tissues samples were embedded in Technovit 7100 (Heraeus Kulzer). Histological slices (3 μm) were obtained by using a microtome Jung RM 2045 Leica. Sections were treated with 0.5% (*w*/*v*) periodic acid and stained with Schiff reagent (20 min; colors polysaccharides in purple) and Naphthol Blue Black (1 min, colors soluble proteins in blue). 

### 4.11. Statistical Analysis

All the experiments were performed by using a completely randomized design (CRD) with three replicates per treatment. In each treatment, 60 explants of leaves and radicles were used. Percentage of callogenesis [(number of explants producing callus per total number of explants], percentage of embryogenic calli [(number of embryogenic calli per number of calli] and mean number of somatic embryos produced per callus (total number of somatic embryos per number of embryogenic calli) were determined. Mean numbers were calculated with their respective standard errors. Data of these observations were analyzed by using standard analysis of variance (ANOVA). The significant difference among treatments was determined using Duncan’s multiple range test at *p* ≤ 0.05.

## 5. Conclusions

The efficiency of in vitro embryogenesis in plants is modulated by many central regulators of regeneration and enhanced by exogenously supplied plant growth regulators. Several key regulators are induced during somatic embryogenesis and control downstream physiological responses to promote callogenesis and embryo production. Our work showed that the expression of the *WUS* gene in *Medicago* explants induced callogenesis and the production of highly embryogenic calli. *WUS*-expressing leaf explants produced embryos and generated plantlets in the absence of growth regulators in the media. This confirmed that the overexpression of the *WUS* gene can be useful for improving tissue culture-based regeneration systems and transformation frequencies of recalcitrant species. Further elucidation of the exact roles of the *WUS* stem cell signaling pathway and of related regulator networks is crucial to understand the diverse strategies of somatic embryogenesis.

## Data Availability

Not applicable.
